# Physical Activity Together for People With Multiple Sclerosis and Their Care Partners: Protocol for a Feasibility Randomized Controlled Trial of a Dyadic Intervention

**DOI:** 10.2196/18410

**Published:** 2021-06-01

**Authors:** Afolasade Fakolade, Julie Cameron, Odessa McKenna, Marcia L Finlayson, Mark S Freedman, Amy E Latimer-Cheung, Lara A Pilutti

**Affiliations:** 1 School of Rehabilitation Therapy Queen's University Kingston, ON Canada; 2 Interdisciplinary School of Health Sciences University of Ottawa Ottawa, ON Canada; 3 The Ottawa Hospital Research Institute Ottawa, ON Canada; 4 Faculty of Medicine University of Ottawa Ottawa, ON Canada; 5 School of Kinesiology and Health Studies Queen's University Kingston, ON Canada

**Keywords:** multiple sclerosis, advanced disability, care partners, physical activity, dyadic intervention, feasibility randomized controlled trial

## Abstract

**Background:**

Physical activity (PA) is beneficial for all people; however, people affected by multiple sclerosis (MS) find regular PA challenging. These people may include individuals with advanced disabilities and their care partners.

**Objective:**

The objective of this study is to determine the feasibility of a dyadic PA intervention for people with advanced MS and their care partners.

**Methods:**

This study is a randomized controlled feasibility trial of a 12-week intervention, with 1:1 allocation into an immediate intervention condition or delayed control condition. A target of 20 people with MS–care partner dyads will be included. The outcomes will be indicators of process, resources, management, and scientific feasibility. Participant satisfaction with the intervention components will be evaluated using a satisfaction survey. The subjective experience of participation in the study will be explored using semistructured interviews.

**Results:**

The project is funded by the Consortium of Multiple Sclerosis Centers. This protocol was approved by the Ottawa Hospital Research Ethics Board (20190329-01H) and the University of Ottawa Research Ethics Board (H-09-19-4886). The study protocol was registered with ClinicalTrials.gov in February 2020. The findings of this feasibility trial will be disseminated through presentations at community events to engage the MS population in the interpretation of our results and in the next steps. The results will also be published in peer-reviewed journals and presented to the scientific community at national and international MS conferences.

**Conclusions:**

The data collected from this feasibility trial will be used to refine the intervention and materials in preparation for a pilot randomized controlled trial.

**Trial Registration:**

ClinicalTrials.gov NCT04267185; https://clinicaltrials.gov/ct2/show/NCT04267185.

**International Registered Report Identifier (IRRID):**

PRR1-10.2196/18410

## Introduction

### Background

Multiple sclerosis (MS) is a neurodegenerative disease characterized by a variable course and largely unpredictable exacerbations leading to progressive disability [1]. Within 15-20 years of disease onset, it is estimated that approximately 50% of people with MS will require a walking aid (eg, cane) to walk about 100 m with or without rest (ie, Expanded Disability Status Scale [2] score≥6) [3]. Approximately 40%-80% of people with MS with walking impairment report a need for support from informal care partners to engage in everyday activities and to participate in aspects of daily life that are important and meaningful to them [4-6]. Care partners are relatives, family members, or friends who provide a broad spectrum of assistance, ranging from help in activities of daily living to emotional support for people with MS [7]. Although there are positive aspects of MS caregiving [8], the negative effect on care partners’ own well-being is often great. MS care partners report poorer quality of life than the general population [9]. These MS care partners also experience a higher level of activity limitations, more emergency department visits, and hospitalizations than care partners of people with other chronic diseases [10]. Together, this evidence suggests that MS has life-altering consequences for people with MS and their care partners and points to an opportunity to identify strategies to improve the health of *both* partners to the benefit of each individual and the dyad (ie, partnership).

Physical activity (PA) is a health-promoting behavior in the general population and an emerging strategy for managing MS [11]. Researchers have reported that PA interventions that incorporate behavioral strategies (eg, goal setting) can increase PA levels and improve symptomatic and participatory outcomes among people with mild-to-moderate MS [12,13]. Emerging evidence from other chronic disease contexts (eg, dementia) suggests that dyadic behavioral PA interventions (ie, targeting *both* care recipients and care partners) can increase PA levels, improve physical and psychological health, and improve exercise adherence for both individuals [14,15]. Other researchers have reported a reduction in stress and improvement in coping skills among care partners of people with dementia after dyadic PA interventions [16,17]. Despite the promise of dyadic PA interventions, no studies to date have capitalized on the potential benefits of including both people with advanced MS and their care partners together as *active participants* in a dyadic behavioral PA intervention. We contend that a dyadic behavioral PA intervention could improve the well-being of people with MS and could help care partners maintain their roles for longer periods with lower health risks.

Within the literature specific to people with MS, there is limited research on behavioral PA interventions that can be widely disseminated for people with advanced MS who experience mobility and transportation limitations [18] and require high levels of caregiving support [19]. Dyadic health researchers have also been challenged to integrate telerehabilitation into intervention design to provide more sustainable and widely disseminated behavioral interventions for dyads with chronic health conditions [20]. Telephone delivery is one of the most widely available telerehabilitation modalities and holds distinct promise for its potential for adoption by public health systems and organizations (eg, MS Societies) that routinely provide telephone support for people with chronic health conditions [21,22]. In particular, group-based teleconferencing offers the added benefit of social modeling, social support, and opportunities for vicarious learning experiences [23,24]. Collectively, existing evidence suggests that telephone-based PA interventions have the potential to increase PA and improve the health of people with MS and their care partners.

### Objectives

The objective of this study is to conduct the first randomized controlled trial (RCT) to determine the feasibility of a dyadic behavioral PA intervention—Physical Activity Together for People With Multiple Sclerosis (PAT-MS) and their care partners. Specifically, we will explore the 4 primary areas of focus of feasibility studies (ie, process, resources, management, and scientific feasibility) [25,26] recommended for PA studies involving people with MS [27].

## Methods

### Study Design

This protocol has been written following both the Standard Protocol Items: Recommendations for Interventional Trials and Consolidated Standards of Reporting Trials guidelines [28,29] (Multimedia Appendix 1). We will conduct a single site, assessor-blinded, parallel group, randomized controlled feasibility trial using a 1:1 allocation into an immediate intervention condition or a delayed intervention condition. A delayed intervention condition was deemed appropriate for PAT-MS based on the decision framework for appropriate control conditions for behavioral intervention trials [30,31].

### Participants

#### Sample Size

As a feasibility trial, this study will provide robust estimates of the likely rates of recruitment and retention as well as estimates of the variability of the proposed scientific outcomes to inform a well-designed pilot RCT. With these considerations in mind, our goal is to recruit 10 people with MS–care partner dyads per condition within a 6-month recruitment window, consistent with sample size guidelines for feasibility trials [32] and previously published exercise trials for people with advanced MS [33]. Enrolling 10 dyads per condition will account for approximately 15% attrition rate, as recommended for feasibility trials [34].

#### Recruitment and Enrolment

Potential participants will be provided with a study information sheet at the local MS clinic and asked for consent to be contacted by the research team. A research coordinator will then contact interested participants by phone to discuss the study and conduct eligibility screening.

The inclusion criteria for people with MS are as follows: (1) a neurologist-confirmed MS diagnosis and stable course of disease-modifying therapies over the past 6 months, (2) an Expanded Disability Status Scale score between 6.0 and 6.5 based on a neurostatus-certified assessor examination, (3) relapse free in the past 30 days, and (4) having a care partner (ie, relative or close friend) who provides ≥1 hour per day of unpaid assistance or help. Additional inclusion criteria for both people with MS and care partners are as follows: (1) ≥18 years of age, (2) currently inactive (ie, purposeful exercise ≤2 days per week for 30 min), and (3) asymptomatic (ie, no major signs or symptoms of acute or uncontrolled cardiovascular, metabolic, or renal disease) based on the Get Active Questionnaire. The exclusion criteria for both people with MS and care partners are (1) presence of other neurological conditions and (2) inability to communicate in English.

### Study Procedures

**Figure 1:**
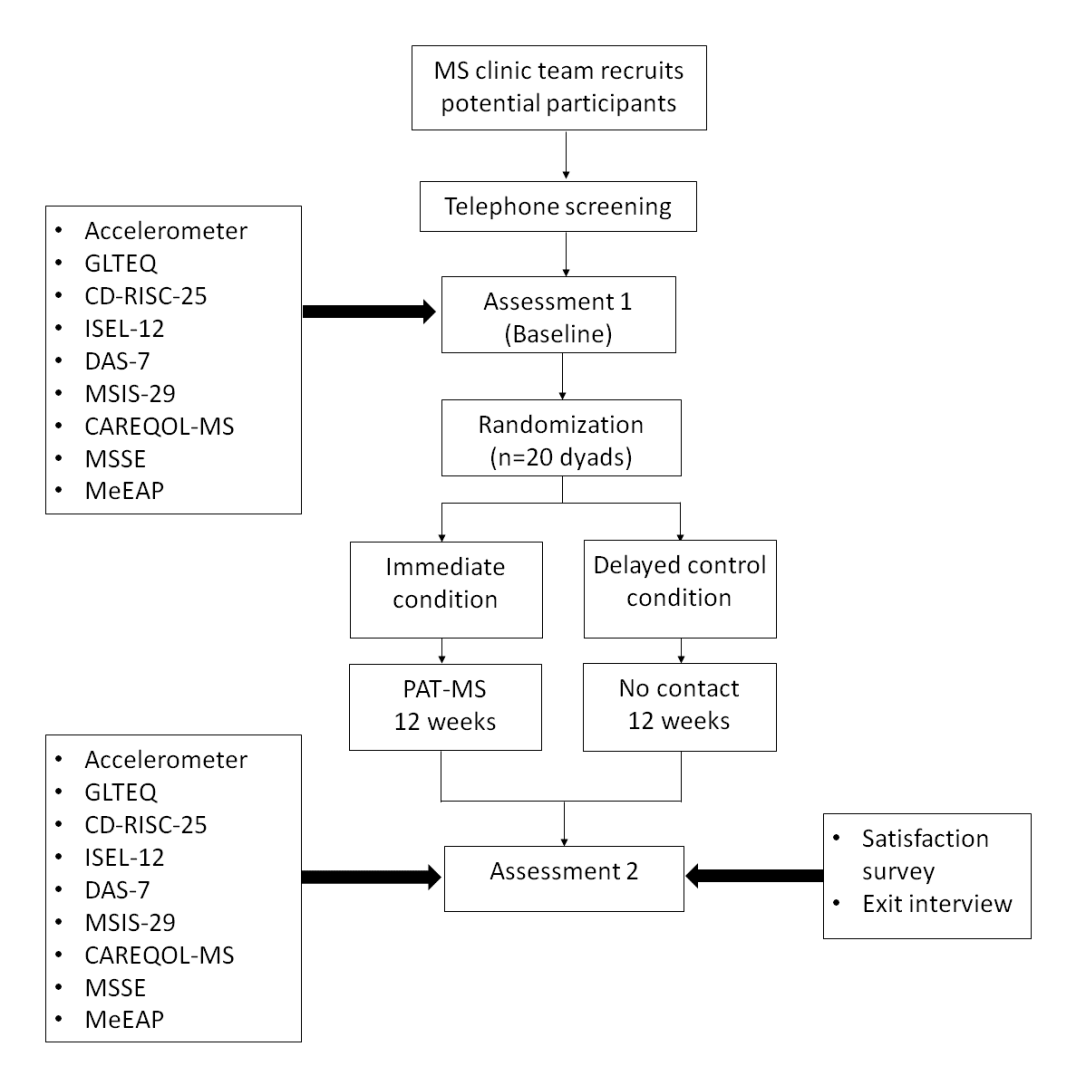
Flow of participants through the study. CAREQOL-MS: Caregiver Quality of Life in Multiple Sclerosis Scale; CD-RISC-10: Connor-Davison Resilience Scale; DAS-7: Short-form Dyadic Adjustment Scale; GLTEQ: Godin Leisure-Time Exercise Questionnaire; ISEL-12: Interpersonal Support Evaluation List-12; MeEAP: Measure of Experiential Aspects of Participation; MS: multiple sclerosis; MSIS-29: Multiple Sclerosis Impact Scale-29; MSSE: Multiple Sclerosis Self-Efficacy Scale; PAT-MS: Physical Activity Together for People With Multiple Sclerosis.

Figure 1 presents the flow of participants throughout the study. All eligible participants will be scheduled for a baseline assessment (T1) for the provision of informed consent and collection of baseline data (scientific feasibility outcomes) at a university research laboratory. People with MS and their care partners will complete the measures in separate rooms to ensure privacy and confidentiality during the data collection process. Outcome measures will be collected by treatment-blinded assessors who are experienced with the administration of the proposed measures. In the 7 days following the baseline assessment, both people with MS and their care partners will be asked to wear an accelerometer during all waking hours. The accelerometer will be placed in a pouch on an elastic belt worn around the waist, with the device placed on the nondominant hip. Prestamped, preaddressed envelopes will be provided for the return of the accelerometer. Randomization will occur after baseline data collection.

The randomization sequence will be generated by an independent biostatistician using 1:1 permutated block randomization. Variable-sized blocks will be used to ensure approximately equal numbers in the 2 trial conditions. Participants will then complete the immediate intervention condition or the delayed intervention condition (ie, maintenance of usual activities) for 12 weeks. The same scientific feasibility outcomes as the baseline will be repeated immediately after the intervention (T2). We anticipate that each assessment session will last for approximately 2 hours. At the completion of the intervention, participants will also be asked to complete a satisfaction survey and an individual exit interview to assess their satisfaction with specific intervention components (ie, intervention content, interventionist, and delivery method) and their subjective experiences of participation in all aspects of PAT-MS, respectively. People with MS and their care partners will complete the same surveys. We anticipate that the survey will take approximately 15 minutes to complete. Individual exit interviews will be conducted over the phone by a member of the research team and will last for approximately 30 minutes. The same interview guide will be used for people with MS and their care partners. Participants in the delayed intervention condition will receive the intervention after the postintervention assessment (T2). We will follow the same intervention delivery procedures for the delayed condition as for the immediate intervention condition.

### Intervention

PAT-MS is a dyadic behavioral PA intervention approach that incorporates a toolbox of evidence-based strategies adapted from previous trials on promoting PA in care partners of people with Alzheimer disease [35,36], a comprehensive program of research [20,37-39], and input from people with MS and care partners. PAT-MS is grounded in the theory of dyadic illness management [40]. Behavior change techniques [41] that target key theoretical constructs from the social cognitive theory [42] and self-determination theory [43] are included to facilitate behavior change. The social cognitive theory and self-determination theory have proven effective for increasing PA in the general population [44] and among people with MS [45-47]. Table 1 provides a summary of the content and main behavior change techniques [41] that will be targeted across the six teleconference sessions. In brief, the intervention includes the following main components:

- Education: Participants will be provided with a PAT-MS manual that includes information on PA and introduces the concepts of shared appraisal of disease impact and dyadic coping. The benefits of shared participation in PA as a coping strategy to optimize well-being at the individual and dyadic levels will be discussed.
- Guidance from a trained interventionist: Participants will be provided with specific verbal and written guidance to improve their confidence in engaging in PA behavior. Given that PA interventions are more effective when combined with behavior change techniques [48], PAT-MS will include techniques commonly used in dyadic health interventions for persons with chronic neurological conditions and their care partners [20]. These techniques include, but are not limited to, goal setting and review, problem solving and action planning, behavioral practice, and instruction from a credible source.
- Social support: Participants will be provided practical, emotional, and informational social support during the intervention (eg, links to community programs for continued PA participation and opportunities to engage with and learn from other group members).

### Intervention Structure and Delivery

People with MS–care partner dyads will receive six group teleconferencing sessions (approximately 60 min each) every other week for a period of 12 weeks (Table 1). Each session is structured to include a review of material from the previous week, teaching content, group discussions, and explanation of practice activities to be completed before the next session. The group sessions will be interspersed with brief (approximately 15 min each) one-on-one support telephone calls in the weeks in which the group sessions do not occur. The intervention schedule will provide regular contact with the research team but will recognize the time commitment of the participants. We will seek to have 2-3 dyads on each teleconference call to manage the call more easily and to monitor the intervention process. The group-based teleconference format will provide opportunities for social modeling, social support, and vicarious learning experiences [23,24], which in turn will support behavior change, consistent with the theoretical foundations of PAT-MS [42,43]. Makeup sessions will be offered to those who miss teleconference sessions. The intervention will be delivered by a trained interventionist who will be provided with a structured manual for intervention delivery. Compliance to the protocol by the interventionist will be monitored using a checklist, weekly review meetings, and episodic monitoring of the teleconference sessions according to the guidelines of Bellg [49].

### Outcomes

The outcomes will relate to process, resources, management, and scientific feasibility, as outlined below.

#### Process Outcomes

Process outcomes will include assessing recruitment rates (ie, response of participants to recruitment strategies, number of potential participants who remain interested in the study after information and screening, and reasons for refusal to participate).

#### Resource Outcomes

Resource outcomes will include assessing the rates of participant compliance (ie, number of practice activities, teleconference sessions, and one-on-one phone calls completed), attrition (ie, percentage of participants who drop out of the study and reasons for dropping out), suitability of eligibility criteria (ie, percentage of interested participants who meet the inclusion criteria and reasons for exclusion), and total cost of intervention delivery (ie, cost of equipment, personnel, and participant remuneration).

#### Management Outcomes

Management outcomes will include assessing staff time (ie, staff preparation and training time, call time, attempted call time, and report-taking time), use of technical support (ie, number of equipment-related data collection problems and number of technical support calls made by staff and/or participants), intervention fidelity (ie, interventionist’s compliance to the protocol), and efficiency and accuracy of data collection and entry (ie, data completeness and time to collect, enter, and check data).

#### Scientific Outcomes

These will include assessing safety, treatment effect, participant satisfaction with intervention components (ie, intervention content, interventionist, and delivery method), and subjective experience of participating in the intervention.

#### Safety

Safety will involve reporting of adverse events (AEs). AEs are defined as any unfavorable change in health experienced by a participant during the trial period [50]. Each AE will be rated based on severity (grade 1 [mild] through 5 [death]), expectedness, and potential relation to study participation (ie, not related, possibly related, or definitely related) using the National Institutes of Health terminology and classification scheme [50]. AEs will be reported as the overall rate, severity, and characteristics of the AEs.

**Table 1:** Timing, content, and main behavior change techniques in Physical Activity Together for People With Multiple Sclerosis.

Timing, description, and overview of session content					Code for behavior change techniques included in each session		Main behavior change techniques included in each session
**Week 1**							
	**Teleconference 1**						
		**Getting started**		2.3		Self-monitoring of behavior	
			Ground rules for the teleconference sessions	3.3		Social support (emotional)	
			Understanding the basics of physical activity	4.1		Instruction on how to perform the behavior	
			How much physical activity do you need?	5.1		Information about health consequences	
			Is it safe for you to participate in physical activity?	5.4		Monitoring of emotional consequences	
			Setting up a physical activity program	9.1		Credible source	
			Recording your baseline physical activity	N/A^a^		N/A	
			Explanation of practice activity	N/A		N/A	
**Week 2**							
	**Phone call 1**						
		Monitoring and providing individualized support, advice, and encouragement		1.2		Problem solving	
		Promoting accountability, maintaining motivation, and troubleshooting		3.2		Social support (practical)	
		N/A		9.1		Credible source	
**Week 3**							
	**Teleconference 2**						
		**Dyadic coping**		1.1		Goal setting (behavior)	
			Review material from last session	1.2		Problem solving	
			Understanding dyadic coping	3.3		Social support (emotional)	
			Setting physical activity goals	4.1		Instruction on how to perform the behavior	
			Setting goals in PAT-MS^b^ physical activity log	8.1		Behavioral practice	
			Explanation of practice activity	9.1		Credible source	
**Week 4**							
	**Phone** **call 2**						
		Monitoring and providing individualized support, advice, and encouragement		1.2		Problem solving	
		Promoting accountability, maintaining motivation, and troubleshooting		3.2		Social support (practical)	
		N/A		9.1		Credible source	
		N/A		10.4		Social reward	
**Week 5**							
	**Teleconference 3**						
		Action planning		1.2		Problem solving	
		Review material from last session		1.4		Action planning	
		What can physical activity do for people with multiple sclerosis?		1.5		Review behavior goals	
		What can physical activity do for support partners?		1.6		Discrepancy between current behavior and goal	
		Developing an action plan for meeting physical activity goals		2.3		Self-monitoring of behavior	
		Explanation of practice activity		5.1		Information about health consequences	
		N/A		8.1		Behavioral practice	
		N/A		8.7		Graded tasks	
		N/A		9.1		Credible source	
		N/A		10.4		Social reward	
**Week 6**							
	**Phone call 3**						
		Monitoring and providing individualized support, advice, and encouragement		1.2		Problem solving	
		Promoting accountability, maintaining motivation, and troubleshooting		3.2		Social support (practical)	
		N/A		9.1		Credible source	
		N/A		10.4		Social reward	
**Week 7**							
	**Teleconference 4**						
		**Staying motivated**		1.2		Problem solving	
			Review material from last session	1.5		Review behavior goals	
			Understanding motivation	1.6		Discrepancy between current behavior and goal	
			Staying motivated to increase your physical activity	2.3		Self-monitoring of behavior	
			Explanation of practice activity	7.1		Prompts or cues	
		N/A		8.1		Behavioral practice	
		N/A		8.7		Graded tasks	
		N/A		9.1		Credible source	
		N/A		10.4		Social reward	
**Week 8**							
	**Phone call 4**						
		Monitoring and providing individualized support, advice, and encouragement		1.2		Problem solving	
		Promoting accountability and troubleshooting		3.2		Social support (practical)	
		N/A		9.1		Credible source	
		N/A		10.4		Social reward	
**Week 9**							
	**Teleconference 5**						
		**Building support**		1.1		Goal setting (behavior)	
			Review material from last session	1.2		Problem solving	
			Building a strong support system	1.5		Review behavior goals	
			Types of social support	1.6		Discrepancy between current behavior and goal	
			Explanation of practice activity	2.3		Self-monitoring of behavior	
		N/A		8.1		Behavioral practice	
		N/A		8.7		Graded tasks	
		N/A		9.1		Credible source	
		N/A		10.4		Social reward	
**Week 10**							
	**Phone call 5**						
		Monitoring and providing individualized support, advice, and encouragement		1.2		Problem solving	
		Promoting accountability and troubleshooting		3.2		Social support (practical)	
		N/A		9.1		Credible source	
		N/A				Social reward	
**Week 11**							
	**Teleconference 6**						
		**Final tips**		1.1		Goal setting (behavior)	
			Review material from last session	1.2		Problem solving	
			Making physical activity a life-long habit	1.5		Review behavior goals	
			Closing comments	9.1		Credible source	
		N/A		9.3		Comparative imagining of future outcomes	
		N/A		10.4		Social reward	
**Week 12**							
	**Phone call 6**						
		Monitoring and providing individualized support, advice, and encouragement		1.2		Problem solving	
		Promoting accountability and troubleshooting		3.2		Social support (practical)	
		N/A		9.1		Credible source	
		N/A		10.4		Social reward	

^a^N/A: not applicable.

^b^PAT-MS: Physical Activity Together for People With Multiple Sclerosis.

#### Treatment Effect

This will involve assessing changes in the following outcomes between T1 (baseline) and T2 (12 weeks): accelerometer-measured PA, self-reported PA, resilience, social support, dyadic relationship quality, quality of life, MS self-efficacy, experiential aspects of participation, and coping. Table 2 provides a summary of the treatment outcomes and psychometric properties of the outcome measures included in the study.

**Table 2:** Treatment effect outcomes, outcome measures, and psychometric properties.

Outcome, outcome measure and psychometric properties				Reliability and validity statistics		Intraclass correlation coefficient
**Change in accelerometer-measured PA^a^ (ie, minutes of sedentary, light, and moderate-to-vigorous activity)**						
	**ActiGraph model GT3X-BT accelerometer [51]**					
		Validity	r_s_=0.61, 95% CI 0.47-0.71		N/A^b^	
		Test-retest reliability	r_s_=0.49, 95% CI 0.40-0.57		0.84, 95% CI 0.81-0.87	
**Change in self-reported PA (ie, total PA minutes)**						
	**Godin Leisure-Time Exercise Questionnaire [52-54]**					
		Convergent validity	*r*=0.38-0.7; *P*<.01		N/A	
		Divergent validity	*r*=−0.32 to −0.45; *P*<.01		N/A	
		Test-retest reliability	*k* coefficient=0.40, 95% CI 0.21-0.60		0.74, 95% CI 0.69-0.78	
**Change in resilience**						
	**Connor-Davison Resilience Scale [55,56]**					
		Internal consistency	Cronbach α=.89		N/A	
		Test-retest reliability	N/A		0.87	
		Convergent validity	*r=*0.83; *P*<.001		N/A	
**Change in social support**						
	**Interpersonal Support Evaluation List-12 [57,58]**					
		Internal consistency	Cronbach α=.82		N/A	
		Convergent validity	*r=*0.33-0.40; *P*<.001		N/A	
**Change in dyadic relationship quality**						
	**Short-form Dyadic Adjustment Scale [59,60]**					
		Internal consistency	Cronbach α=.78		N/A	
		Construct validity	*r*=0.38-0.72; *P*<.01		N/A	
**Change in quality of life among people with MS^c^**						
	**Multiple Sclerosis Impact Scale-29 [61,62]**					
		Reliability	Cronbach α≥.89 for all subscales		N/A	
		Convergent validity	r_s_≥0.57; *P*<.001		N/A	
**Change in quality of life among MS care partners**						
	**Caregiver Quality of Life in Multiple Sclerosis Scale [63]**					
		Internal consistency	Cronbach α≥.75 for all subscales		N/A	
		Test-retest reliability	Weighted *k*≥0.46		N/A	
		Construct validity	N/A		0.96; *P*<.001	
**Change in MS self-efficacy**						
	**Multiple Sclerosis Self-Efficacy Scale [64]**					
		Internal consistency	Cronbach α=.81		N/A	
		Test-retest reliability	*r*=0.81; *P*<.001		N/A	
		Construct validity	r_s_≥0.55; *P*<.01		N/A	
**Change in experiential aspects of participation**						
	**Measure of Experiential Aspects of Participation [65]**					
		Internal consistency	Cronbach α≥.95 for all subscales		N/A	
		Convergent validity	N/A		≥0.62; *P*<.001 for all subscales	
**Change in coping among MS care partners**						
	**Coping with Multiple Sclerosis Caregiving Inventory [66]**					
		Internal consistency	Cronbach α≥.57 for all subscales		N/A	

^a^PA: physical activity.

^b^N/A: not applicable.

^c^MS: multiple sclerosis.

#### Satisfaction With Intervention Components—Content, Interventionist, and Delivery Method

This will be assessed in both people with MS and care partners using a satisfaction survey developed for this study. Items will be scored using a 5-point Likert-type scale, with higher scores reflecting greater satisfaction.

#### Subjective Experience of Participation in All Aspects of PAT-MS

This will be explored in both people with MS and care partners using a semistructured exit interview. Suggestions for intervention improvement and participants’ willingness and concerns regarding future participation in PA will also be explored.

### Data Management and Analysis

#### Quantitative Data Analysis

Data management and analysis will be performed using IBM SPSS Statistics for Windows (IBM Corp). Descriptive statistics, including means and SDs (continuous variables) and frequencies and proportions (categorical variables), will be used to summarize all demographic and feasibility data. Within-subject changes and effect sizes for improvement in scientific outcomes from T1 to T2 will be calculated using Cohen *d* separately for people with MS and care partners and by condition (immediate vs delayed control).

#### Qualitative Data Analysis

All audiorecorded interviews will be transcribed and anonymized before the analysis. The qualitative analysis will be underpinned by a social constructivism paradigm [67], which will allow exploration of the meaning and understanding of the experiences of our participants relative to participation in PAT-MS. The systematic six-phase process of thematic analysis as described by Braun and Clark [68] will be undertaken. The rigor of the qualitative analysis will be maximized through a range of strategies recommended by Smith and McGannon [69].

### Determining Progression to a Definitive Trial

Progression to a pilot RCT will be considered if minimum success criteria are achieved in key feasibility metrics or if we can identify strategies for overcoming any identified challenges in these areas [70]. These criteria were selected based on the guidelines for prospectively defining progression to future evaluative studies [71,72]. The criteria include the following:

- A minimum of 50% of the intended 20 dyads are recruited within a 6-month recruitment window
- A minimum of 70% participant compliance
- Study satisfaction ≥4/5 on the satisfaction survey
- Less than 10% of participants report a serious AE
- Less than 20% participant attrition.

### Ethics Approval and Consent to Participate

This protocol was approved by the Ottawa Hospital Research Ethics Board (20190329-01H) and the University of Ottawa Research Ethics Board (H-09-19-4886). The trial is conducted in compliance with the Declaration of Helsinki. Informed written consent will be obtained from all the participants.

## Results

The project is funded by the Consortium of Multiple Sclerosis Centers. This protocol was approved by the Ottawa Hospital Research Ethics Board (20190329-01H) and the University of Ottawa Research Ethics Board (H-09-19-4886). The study protocol was registered with ClinicalTrials.gov (NCT04267185) in February 2020. The findings of this feasibility trial will be disseminated through presentations at community events to engage the MS population in the interpretation of our results and in the next steps. The results will also be published in peer-reviewed journals and presented to the scientific community at national and international MS conferences.

## Discussion

### Principal Findings

Several direct outcomes are anticipated from this trial. First, the delivery of this trial will provide important insights for the research team on the practicality of running a future pilot trial, if the proposed intervention is feasible. Second, this trial will provide key information on the feasibility of PAT-MS, including estimates of recruitment, compliance, and attrition. It will also enable us to assess the acceptability of the intervention from the participants’ perspective. Finally, conducting this work will lead to the development of a manualized research protocol for PAT-MS. This manual will include the recruitment and selection criteria, details about the intervention and training of intervention staff, recommendations for managing study logistics, and possible challenges and strategies for overcoming them. The development of this manual will facilitate the delivery of future efficacy and effectiveness trials, including those using a multicenter approach. In addition, it will ensure the fidelity of the intervention and its long-term delivery in community, health care, or other multiservice settings.

### Strengths and Limitations

The PAT-MS intervention is unique in several ways. PAT-MS uses a novel approach that combines both people with MS and their care partners together as active and collaborative participants in the intervention. There are potential synergistic benefits of this intervention on the health of each partner individually and on the dyad (ie, partnership). In addition, the focus on people with advanced MS disability is novel, as few interventions target this segment of the MS population. This MS cohort requires accessible strategies for disease management and requires high levels of caregiving support. Finally, the use of a telerehabilitation offers a cost-effective strategy for widespread long-term dissemination and is one of the preferred delivery formats for PA interventions among people with MS [73].

When executing the proposed trial, foreseeable challenges are compliance and attrition, which may be related to disease symptoms, comorbidities, or changes in medications. Care partners who are often working outside of the home, in addition to their caregiving role, may perceive participating in the intervention as a dyad to be burdensome, rather than beneficial. We have incorporated various methods into the study design to maximize retention and compliance: (1) flexibility in assessment sessions and phone call times; (2) follow-up by telephone and makeup session options; (3) offsetting participation costs through remuneration, toll-free calling, and reserved parking for testing visits; (4) including intervention content on the potential benefits of regular PA participation and how participants can safely engage in PA; and (5) incorporating behavior change techniques and using group-based delivery to reinforce social support, social modeling, and vicarious learning. Another potential challenge is the fidelity of the intervention. To promote the standard application of the intervention, an interventionist manual will be provided and incorporated into the interventionist’s training.

### Conclusions

This is the first study to examine the feasibility of the PAT-MS intervention. PAT-MS offers people with MS who have advanced disability and their care partners an opportunity to achieve important health and well-being benefits associated with PA participation. The findings from this study will be relevant in informing future dyadic health promotion research in MS.

## Appendix

Multimedia Appendix 1 CONSORT (Consolidated Standards of Reporting Trials) checklist.

## Abbreviations

AE: adverse event
MS: multiple sclerosis
PA: physical activity
PAT-MS: Physical Activity Together for People With Multiple Sclerosis
RCT: randomized controlled trial

## References

[1] Kesselring J, Beer S (2005). Symptomatic therapy and neurorehabilitation in multiple sclerosis. Lancet Neurol.

[2] Hohol M, Orav E, Weiner HL (1999). Disease steps in multiple sclerosis: a longitudinal study comparing disease steps and EDSS to evaluate disease progression. Mult Scler.

[3] Tremlett H, Paty D, Devonshire V (2006). Disability progression in multiple sclerosis is slower than previously reported. Neurology.

[4] Holland NJ, Northrop DE (2016). Young adults with multiple sclerosis: management in the home. Home Health Care Manag Pract.

[5] Hillman L (2013). Caregiving in multiple sclerosis. Phys Med Rehabil Clin N Am.

[6] Larocca NG (2011). Impact of walking impairment in multiple sclerosis: perspectives of patients and care partners. Patient.

[7] Finlayson M, Cho C (2008). A descriptive profile of caregivers of older adults with MS and the assistance they provide. Disabil Rehabil.

[8] Pakenham KI (2005). Benefit finding in multiple sclerosis and associations with positive and negative outcomes. Health Psychol.

[9] Patti F, Amato MP, Battaglia MA, Pitaro M, Russo P, Solaro C, Trojano M (2007). Caregiver quality of life in multiple sclerosis: a multicentre Italian study. Mult Scler.

[10] Gupta S, Goren A, Phillips AL, Stewart M (2012). Self-reported burden among caregivers of patients with multiple sclerosis. Int J MS Care.

[11] Motl RW (2014). Lifestyle physical activity in persons with multiple sclerosis: the new kid on the MS block. Mult Scler.

[12] Bombardier CH, Cunniffe M, Wadhwani R, Gibbons LE, Blake KD, Kraft GH (2008). The efficacy of telephone counseling for health promotion in people with multiple sclerosis: a randomized controlled trial. Arch Phys Med Rehabil.

[13] Motl RW, Hubbard EA, Bollaert RE, Adamson BC, Kinnett-Hopkins D, Balto JM, Sommer SK, Pilutti LA, McAuley E (2017). Randomized controlled trial of an e-learning designed behavioral intervention for increasing physical activity behavior in multiple sclerosis. Mult Scler J Exp Transl Clin.

[14] Prick A-E, de Lange J, Scherder E, Twisk J, Pot AM (2016). The effects of a multicomponent dyadic intervention on the mood, behavior, and physical health of people with dementia: a randomized controlled trial. Clin Interv Aging.

[15] Prick AE, de Lange J, Twisk J, Pot AM (2015). The effects of a multi-component dyadic intervention on the psychological distress of family caregivers providing care to people with dementia: a randomized controlled trial. Int Psychogeriatr.

[16] Canonici AP, Andrade LP, Gobbi S, Santos-Galduroz RF, Gobbi LT, Stella F (2012). Functional dependence and caregiver burden in Alzheimer's disease: a controlled trial on the benefits of motor intervention. Psychogeriatrics.

[17] Lowery D, Cerga-Pashoja A, Iliffe S, Thuné-Boyle I, Griffin M, Lee J, Bailey A, Bhattacharya R, Warner J (2014). The effect of exercise on behavioural and psychological symptoms of dementia: the EVIDEM-E randomised controlled clinical trial. Int J Geriatr Psychiatry.

[18] Chiu C, Bishop M, Pionke J, Strauser D, Santens RL (2017). Barriers to the accessibility and continuity of health-care services in people with multiple sclerosis: a literature review. Int J MS Care.

[19] Buchanan RJ, Radin D, Chakravorty BJ, Tyry T (2009). Informal care giving to more disabled people with multiple sclerosis. Disabil Rehabil.

[20] Fakolade A, Walters AJ, Cameron J, Latimer-Cheung AE, Pilutti LA (2020). Healthy together: a systematic review of theory and techniques used in health interventions for persons with chronic neurological conditions and their caregivers. Patient Educ Couns.

[21] Castro CM, King AC (2002). Telephone-assisted counseling for physical activity. Exerc Sport Sci Rev.

[22] Goode AD, Reeves MM, Eakin EG (2012). Telephone-delivered interventions for physical activity and dietary behavior change: an updated systematic review. Am J Prev Med.

[23] Fakolade A, Finlayson M, Plow M (2017). Using telerehabilitation to support people with multiple sclerosis: a qualitative analysis of interactions, processes, and issues across three interventions. Br J Occup Ther.

[24] Borek AJ, Abraham C, Greaves CJ, Gillison F, Tarrant M, Morgan-Trimmer S, McCabe R, Smith JR (2019). Identifying change processes in group-based health behaviour-change interventions: development of the mechanisms of action in group-based interventions (MAGI) framework. Health Psychol Rev.

[25] Thabane L, Ma J, Chu R, Cheng J, Ismaila A, Rios LP, Robson R, Thabane M, Giangregorio L, Goldsmith CH (2010). A tutorial on pilot studies: the what, why and how. BMC Med Res Methodol.

[26] Tickle-Degnen L (2013). Nuts and bolts of conducting feasibility studies. Am J Occup Ther.

[27] Learmonth YC, Motl RW (2018). Important considerations for feasibility studies in physical activity research involving persons with multiple sclerosis: a scoping systematic review and case study. Pilot Feasibility Stud.

[28] Eldridge SM, Chan CL, Campbell MJ, Bond CM, Hopewell S, Thabane L, Lancaster GA, PAFS consensus group (2016). Consort 2010 statement: extension to randomised pilot and feasibility trials. Br Med J.

[29] Chan AW, Tetzlaff J, Altman DG, Laupacis A, Gøtzsche PC, Krleža-Jerić K, Hróbjartsson A, Mann H, Dickersin K, Berlin JA, Doré CJ, Parulekar WR, Summerskill WS, Groves T, Schulz KF, Sox HC, Rockhold FW, Rennie D, Moher D (2013). Spirit 2013 statement: defining standard protocol items for clinical trials. Ann Intern Med.

[30] Gold SM, Enck P, Hasselmann H, Friede T, Hegerl U, Mohr DC, Otte C (2017). Control conditions for randomised trials of behavioural interventions in psychiatry: a decision framework. Lancet Psychiatry.

[31] Bamman MM, Cutter GR, Brienza DM, Chae J, Corcos DM, DeLuca S, Field-Fote E, Fouad MN, Lang CE, Lindblad A, Motl RW, Perna CG, Reisman D, Saag KM, Savitz SI, Schmitz KH, Stevens-Lapsley J, Whyte J, Winstein CJ, Michel ME (2018). Medical rehabilitation: guidelines to advance the field with high-impact clinical trials. Arch Phys Med Rehabil.

[32] Hertzog MA (2008). Considerations in determining sample size for pilot studies. Res Nurs Health.

[33] Edwards T, Pilutti LA (2017). The effect of exercise training in adults with multiple sclerosis with severe mobility disability: a systematic review and future research directions. Mult Scler Relat Disord.

[34] Browne RH (1995). On the use of a pilot sample for sample size determination. Stat Med.

[35] Farran CJ, Etkin CD, Eisenstein A, Paun O, Rajan KB, Sweet CM, McCann JJ, Barnes LL, Shah RC, Evans DA (2016). Effect of moderate to vigorous physical activity intervention on improving dementia family caregiver physical function: a randomized controlled trial. J Alzheimers Dis Parkinsonism.

[36] Farran CJ, Staffileno BA, Gilley DW, McCann JJ, Li Y, Castro CM, King AC (2008). A lifestyle physical activity intervention for caregivers of persons with Alzheimer's disease. Am J Alzheimers Dis Other Demen.

[37] Fakolade A, Lamarre J, Latimer-Cheung A, Parsons T, Morrow SA, Finlayson M (2018). Understanding leisure-time physical activity: voices of people with MS who have moderate-to-severe disability and their family caregivers. Health Expect.

[38] Fakolade A, Finlayson M, Parsons T, Latimer-Cheung A (2018). Correlating the physical activity patterns of people with moderate to severe multiple sclerosis disability and their family caregivers. Physiother Can.

[39] Fakolade A, Latimer-Cheung A, Parsons T, Finlayson M (2019). A concerns report survey of physical activity support needs of people with moderate-to-severe MS disability and family caregivers. Disabil Rehabil.

[40] Lyons KS, Lee CS (2018). The theory of dyadic illness management. J Fam Nurs.

[41] Michie S, Richardson M, Johnston M, Abraham C, Francis J, Hardeman W, Eccles MP, Cane J, Wood CE (2013). The behavior change technique taxonomy (v1) of 93 hierarchically clustered techniques: building an international consensus for the reporting of behavior change interventions. Ann Behav Med.

[42] Bandura A (2001). Social cognitive theory: an agentic perspective. Annu Rev Psychol.

[43] Deci EL, Ryan RM (2008). Self-determination theory: a macrotheory of human motivation, development, and health. Can Psychol.

[44] Fortier MS, Duda JL, Guerin E, Teixeira PJ (2012). Promoting physical activity: development and testing of self-determination theory-based interventions. Int J Behav Nutr Phys Act.

[45] Bombardier CH, Ehde DM, Gibbons LE, Wadhwani R, Sullivan MD, Rosenberg DE, Kraft GH (2013). Telephone-based physical activity counseling for major depression in people with multiple sclerosis. J Consult Clin Psychol.

[46] Pilutti LA, Dlugonski D, Sandroff BM, Klaren R, Motl RW (2014). Randomized controlled trial of a behavioral intervention targeting symptoms and physical activity in multiple sclerosis. Mult Scler.

[47] Sandroff BM, Klaren RE, Pilutti LA, Dlugonski D, Benedict RH, Motl RW (2014). Randomized controlled trial of physical activity, cognition, and walking in multiple sclerosis. J Neurol.

[48] Michie S (2008). Designing and implementing behaviour change interventions to improve population health. J Health Serv Res Policy.

[49] Bellg AJ, Borrelli B, Resnick B, Hecht J, Minicucci DS, Ory M, Ogedegbe G, Orwig D, Ernst D, Czajkowski S, Treatment Fidelity Workgroup of the NIH Behavior Change Consortium (2004). Enhancing treatment fidelity in health behavior change studies: best practices and recommendations from the NIH Behavior Change Consortium. Health Psychol.

[50] (2017). Common Terminology Criteria for Adverse Events (CTCAE) Version 5.0. U.S. Department of Health and Human Services.

[51] Motl RW, McAuley E, Klaren R (2014). Reliability of physical-activity measures over six months in adults with multiple sclerosis: implications for designing behavioral interventions. Behav Med.

[52] Godin G, Sheperd RJ (1985). A simple method to assess exercise behavior in the community. Can J Appl Sport Sci.

[53] Amireault S, Godin G (2015). The Godin-Shephard Leisure-Time Physical Activity Questionnaire: validity evidence supporting its use for classifying healthy adults into active and insufficiently active categories. Percept Mot Skills.

[54] Sikes EM, Richardson EV, Cederberg KJ, Sasaki JE, Sandroff BM, Motl RW (2019). Use of the Godin leisure-time exercise questionnaire in multiple sclerosis research: a comprehensive narrative review. Disabil Rehabil.

[55] Connor KM, Davidson JR (2003). Development of a new resilience scale: the Connor-Davidson Resilience Scale (CD-RISC). Depress Anxiety.

[56] Windle G, Bennett KM, Noyes J (2011). A methodological review of resilience measurement scales. Health Qual Life Outcomes.

[57] Cohen S, Hoberman HM (1983). Positive events and social supports as buffers of life change stress. J Appl Social Pyschol.

[58] Merz EL, Roesch SC, Malcarne VL, Penedo FJ, Llabre MM, Weitzman OB, Navas-Nacher EL, Perreira KM, Gonzalez F, Ponguta LA, Johnson TP, Gallo LC (2014). Validation of interpersonal support evaluation list-12 (ISEL-12) scores among English- and Spanish-speaking Hispanics/Latinos from the HCHS/SOL Sociocultural Ancillary Study. Psychol Assess.

[59] Spanier GB (1976). Measuring dyadic adjustment: new scales for assessing the quality of marriage and similar dyads. J Marriage Fam.

[60] Hunsley J, Best M, Lefebvre M, Vito D (2001). The seven-item short form of the Dyadic Adjustment Scale: further evidence for construct validity. Am J Fam Ther.

[61] Hobart J, Lamping D, Fitzpatrick R, Riazi A, Thompson A (2001). The Multiple Sclerosis Impact Scale (MSIS-29): a new patient-based outcome measure. Brain.

[62] McGuigan C, Hutchinson M (2004). The multiple sclerosis impact scale (MSIS-29) is a reliable and sensitive measure. J Neurol Neurosurg Psychiatry.

[63] Benito-León J, Rivera-Navarro J, Guerrero AL, de Las Heras V, Balseiro J, Rodríguez E, Belló M, Martínez-Martín P, Caregiver Quality Of Life in Multiple Sclerosis (CAREQOL-MS) Study Group (2011). The CAREQOL-MS was a useful instrument to measure caregiver quality of life in multiple sclerosis. J Clin Epidemiol.

[64] Rigby SA, Domenech C, Thornton EW, Tedman S, Young CA (2003). Development and validation of a self-efficacy measure for people with multiple sclerosis: the Multiple Sclerosis Self-efficacy Scale. Mult Scler.

[65] Caron JG, Ginis KA, Rocchi M, Sweet SN (2019). Development of the measure of experiential aspects of participation for people with physical disabilities. Arch Phys Med Rehabil.

[66] Pakenham K (2002). Development of a measure of coping with multiple sclerosis caregiving. Psychol Health.

[67] Creswell JW (2012). Qualitative Inquiry and Research Design: Choosing Among Five Approaches.

[68] Braun V, Clarke V (2019). Reflecting on reflexive thematic analysis. Qual Res Sport Exerc Health.

[69] Smith B, McGannon KR (2017). Developing rigor in qualitative research: problems and opportunities within sport and exercise psychology. Int Rev Sport Exerc Psychol.

[70] Hallingberg B, Turley R, Segrott J, Wight D, Craig P, Moore L, Murphy S, Robling M, Simpson SA, Moore G (2018). Exploratory studies to decide whether and how to proceed with full-scale evaluations of public health interventions: a systematic review of guidance. Pilot Feasibility Stud.

[71] Avery KN, Williamson PR, Gamble C, Francischetto EO, Metcalfe C, Davidson P, Williams H, Blazeby JM, Members of the Internal Pilot Trials Workshop supported by the Hubs for Trials Methodology Research (2017). Informing efficient randomised controlled trials: exploration of challenges in developing progression criteria for internal pilot studies. BMJ Open.

[72] Young HM, Goodliffe S, Madhani M, Phelps K, Regen E, Locke A, Burton JO, Singh SJ, Smith AC, Conroy S (2019). Co-producing progression criteria for feasibility studies: a partnership between patient contributors, clinicians and researchers. Int J Environ Res Public Health.

[73] Sweet SN, Perrier MJ, Podzyhun C, Latimer-Cheung AE (2013). Identifying physical activity information needs and preferred methods of delivery of people with multiple sclerosis. Disabil Rehabil.

